# Spikelets and bursts in axonless retinal AII amacrine cells coupled by gap junctions

**DOI:** 10.1186/1471-2202-14-S1-P364

**Published:** 2013-07-08

**Authors:** Hermann Riecke, Hannah Choi, Mark S Cembrowski, William L Kath, Joshua H Singer

**Affiliations:** 1 Applied Mathematics, Northwestern University, Evanston, IL 60208, USA; 2 HHMI Janelia Farm Research Campus, Ashburn, VA 20147, USA; 3 Dept. of Biology, U. Maryland, College Park, MD 20742, USA

## 

AII amacrine cells play a central role in the processing of visual signals in the retina. They do not have axons but possess elaborate, ramified dendrites, through which they receive low-light input from rod bipolar cells. These dendrites also serve as output structures: chemical synapses inhibit the OFF cone-pathway and gap junctions provide input into the ON cone-pathway. Gap junctions also directly couple the AIIs to one another. In slice experiments, the AIIs are found to generate surprisingly small action potentials and can exhibit persistent bursting. In mouse rd1-mutants, which serve as a model for retinal degeneration, the death of the photoreceptors is followed by the appearance of strong rhythmicity in the spiking activity of the ganglion cells, which provide the output of the retina.

**Figure 1:**
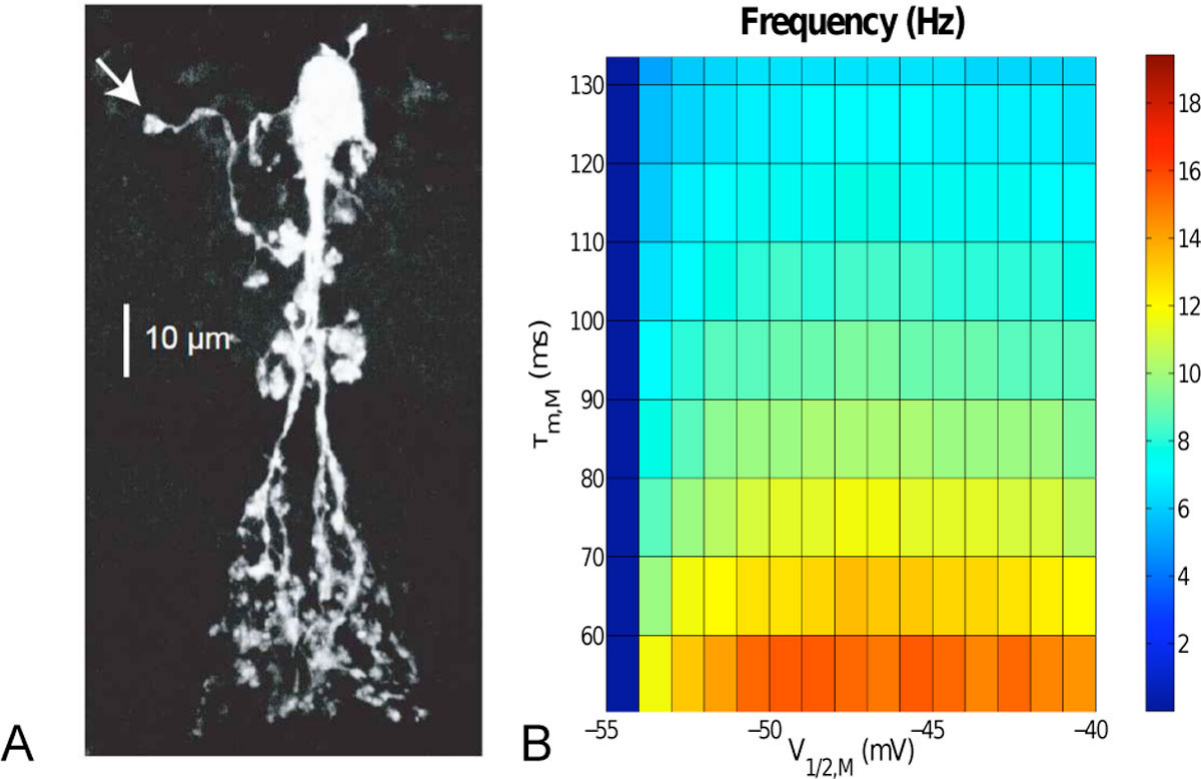
**A Confocal image of an AII**. Putative spike initiation site is marked by arrow. B Dependence of the burst frequency on M-current kinetics (half-activation voltage and deactivation time).

Using computational modeling we show that the small size of the action potentials, as observed in the soma, is due to the ramified morphology of the AII (Figure 1A). In fact, the modeling reveals that the spikes must originate in a *single *compartment that is electrotonically distant from the soma. Our modeling and subsequent electrophysiology experiments show that the bursting is due to a slow current, most likely an M-type K-conductance [1].

Experimentally, it has been found that the oscillations in rd1-mutant mice are abolished by the gap-junction blocker MFA [2]. Our model suggests that the elimination of the oscillations is not due to the blocking of the gap junctions but rather by the known effects of MFA on the kinetics of the M-type K-current: MFA shifts the activation curve of the M-current to more negative potentials and slows down its deactivation [3]. Consistent with experiments [2] our model shows a decrease in oscillation frequency before the oscillations are abolished with increasing MFA concentration (Figure 1B). Moreover, it generates tonic firing if the AIIs are depolarized in the presence of MFA, as has been observed experimentally [4].
